# A “Watchful Surgery” Approach: Timing and Valve Choice for Mildly Symptomatic Mitral and Aortic Valve Disease

**DOI:** 10.26502/jsr.10020472

**Published:** 2025-09-22

**Authors:** Shaanali Mukadam, Chang Kon Kim, Devendra K. Agrawal

**Affiliations:** Department of Translational Research, College of Osteopathic Medicine of the Pacific, Western University of Health Sciences, Pomona, California 91766 USA

**Keywords:** Aortic valve insufficiency, Aortic valve stenosis, Early surgical intervention, Heart valve prosthesis, Mitral regurgitation, Mitral valve insufficiency, Mitral valve stenosis, Surgical aortic valve replacement (SAVR), Transcatheter Aortic valve replacement (TAVR), Valve-in-valve procedures, Ventricular remodeling

## Abstract

The timing of surgical intervention and prosthesis selection in mildly symptomatic aortic and mitral valve disease remain an area of clinical uncertainty. Symptom-based referral often occurs after the onset of adverse ventricular remodeling, whereas earlier surgery may improve long-term outcomes but introduces procedural risk. This article synthesized contemporary evidence to delineate optimal strategies in this intermediate-risk population. In aortic stenosis, randomized trials including RECOVERY and AVATAR demonstrate that early surgical aortic valve replacement reduces heart failure events and may improve survival. In aortic and mitral regurgitation, observational data associate early intervention, triggered by mild symptoms or subclinical ventricular dysfunction, with improved survival and preservation of cardiac function. Emerging modalities such as global longitudinal strain and natriuretic peptide biomarkers enhance risk stratification in asymptomatic individuals. Prosthesis selection is primarily informed by patient age, comorbidity burden, and anticoagulation tolerance. Mechanical valves confer superior durability and lower reoperation risk in younger patients. Bioprosthetic valves are increasingly favored in older adults due to compatibility with transcatheter valve-in-valve reintervention and avoidance of lifelong anticoagulation. Comparative cohort studies and meta-analyses suggest a survival benefit for mechanical valves up to approximately 65-to-70 years of age, beyond which the benefit diminishes due to competing mortality risks. Evidence supports timely surgical referral in mildly symptomatic patients, particularly in the presence of early imaging or biomarker evidence of ventricular dysfunction. Individualized decision-making through multidisciplinary heart team evaluation remains essential. Further investigations are warranted to define long-term prosthetic durability and the role of early surgery in valvular regurgitation.

## 1. Introduction

**Figure 1:**
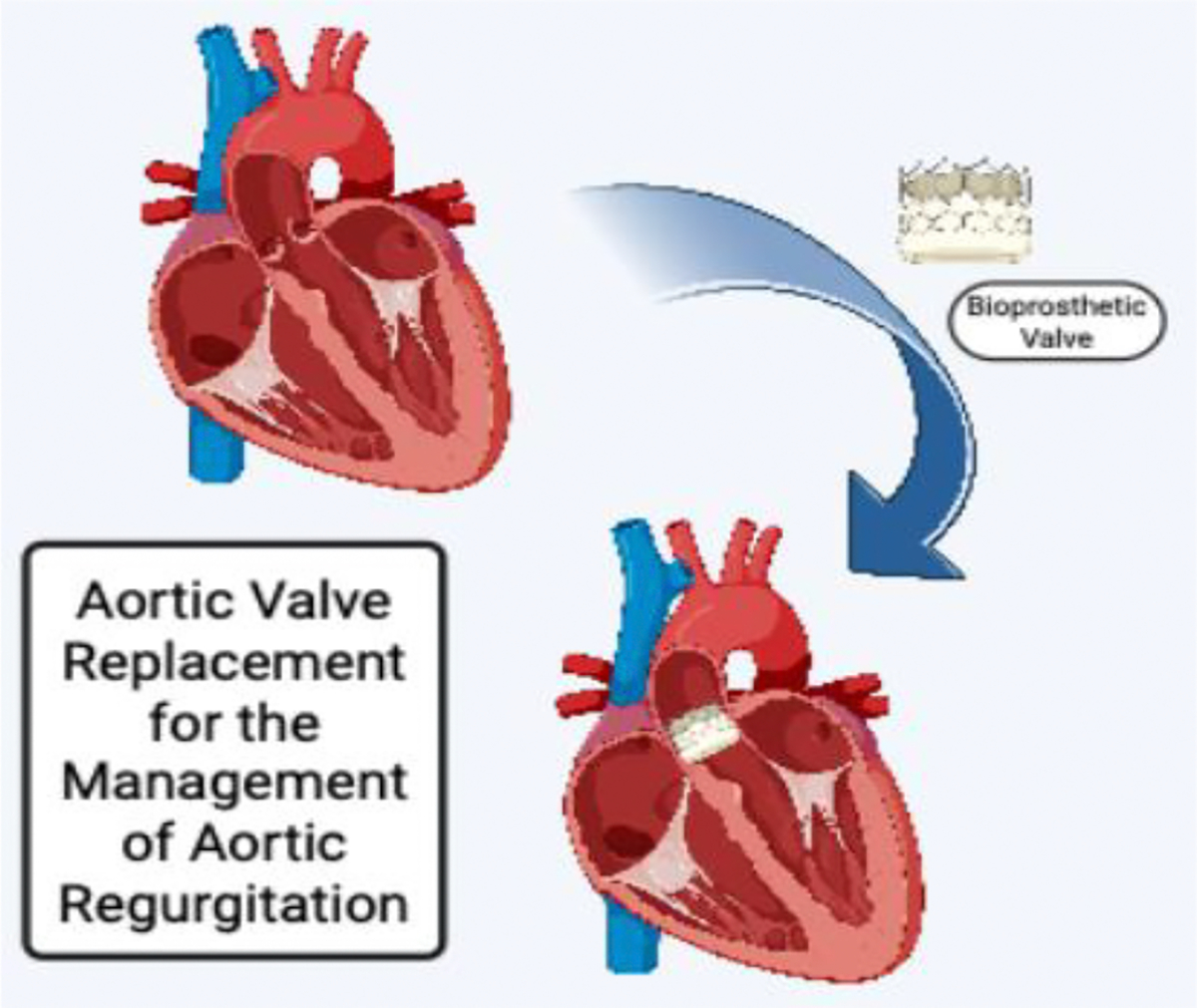
Conceptual illustration of aortic valve replacement (AVR) for chronic aortic regurgitation, created with BioRender. The image depicts transition from a native aortic valve with regurgitation to a surgically implanted bioprosthetic valve, representing correction of valvular insufficiency and restoration of forward flow.

Valvular heart disease (VHD) refers to structural pathology of the cardiac valves. In adults, the most clinically significant lesions are aortic stenosis (AS), aortic regurgitation (AR), and mitral regurgitation (MR), which represent the predominant indications for surgical valve replacement (Figure 1). Degenerative AS is now the most common valvular lesion in high-income countries, with prevalence rising steeply with age [1]. In elderly cohorts, clinically relevant valve lesions are frequently encountered. Severe AR affects approximately 2.0–2.5% of adults aged 70 to 83 years [1], and moderate or greater MR is identified in about 3.5% of older adults undergoing screening echocardiography [2]. One large population study found that more than 70% of individuals over age 65 had some form of valvular abnormality, most commonly mild and clinically silent [3]. Although early or mild VHD may have limited clinical significance, advanced disease contributes substantially to morbidity and mortality. Contemporary U.S. data indicate that VHD accounts for approximately 0.8% of all deaths and 2.4% of cardiovascular deaths, with AS representing the single most common valvular cause [4]. Given the aging of the population, the burden of aortic and mitral valve disease is expected to rise, underscoring the need for optimized strategies in timing and management [1, 4].

## 2. Importance of early diagnosis and treatment

Because valvular lesions often progress insidiously, timely identification and intervention are essential. Advances in surveillance and treatment over the past two decades have coincided with a reduction in VHD-related mortality [4]. Nonetheless, underdiagnosis remains common. In one community screening of older adults, nearly half of moderate or greater MR cases were previously undiagnosed, and only 2.4% of affected patients underwent surgery during approximately five years of follow-up [2]. The natural history of untreated VHD is frequently unfavorable. In a national echocardiographic registry of over 600,000 patients, conservative management of severe MR was associated with significantly lower survival [5]. Similarly, observational data suggest a survival benefit to timely surgery. In older adults with severe AR and mild symptoms, aortic valve replacement (AVR) was associated with significantly lower all-cause and cardiac mortality compared to conservative management [1]. Delaying surgery in severe valvular disease risks irreversible ventricular remodeling, affirming the need for early detection and prompt intervention [1,4].

## 3. Timing of surgery in mildly symptomatic patients

Determining the optimal timing of valve intervention in patients with mild symptoms remains a significant clinical challenge. Current guidelines generally recommend surgery for severe valvular heart disease once overt symptoms or evidence of ventricular dysfunction emerges [6, 7]. However, this symptom-guided approach is increasingly questioned, particularly in patients at high risk for adverse remodeling or in those whose symptom burden may be underrecognized.

Recent studies have begun to challenge the traditional paradigm [1, 8, 9]. In patients with severe aortic regurgitation and mild symptoms, early aortic valve replacement has been associated with improved survival [1]. Similar findings have been reported in aortic stenosis; randomized trials such as AVATAR and RECOVERY suggest that early surgical intervention may reduce heart failure events and mortality, although longer-term data are still maturing [8,9]. Conversely, large observational series consistently report high mortality among patients with untreated severe AS or MR, underscoring the potential harm of delayed referral [8, 10, 11]. As the evidence base continues to evolve, the timing of surgery in mildly symptomatic patients remains an area of active investigation. This review examines the most recent clinical data comparing early versus delayed surgical intervention in this population.

## 4. Prosthesis selection: mechanical versus bioprosthetic valves

Once surgical intervention is indicated, prosthesis selection introduces additional clinical considerations. Mechanical valves offer superior long-term durability but necessitate lifelong anticoagulation, whereas bioprosthetic valves eliminate the need for chronic anticoagulation but are subject to structural valve degeneration. Contemporary cohort studies have characterized this tradeoff across age strata. Mechanical prostheses have been associated with improved long-term survival in patients undergoing aortic valve replacement up to approximately 65 years of age, and in those undergoing mitral valve replacement up to around 70 years [12]. Beyond these thresholds, the survival advantage diminishes, likely reflecting the increased risk of anticoagulation-related complications in older adults. Additional multicenter analyses have demonstrated no significant survival difference between valve types in patients over 50 years old, although younger patients receiving bioprostheses experience higher rates of reoperation[13]. Patient age remains a key determinant of prosthesis selection, with younger individuals deriving benefit from the enhanced durability of mechanical valves, while older adults more commonly favor bioprosthetic options to mitigate anticoagulation-related risk [12, 13]. This clinical decision-making framework, including long-term reintervention and bleeding risk, is examined in greater detail in subsequent sections of this review.

## 5. Purpose and scope of the article

This article critically evaluates contemporary primary research findings on the timing of surgical valve replacement and prosthesis selection in patients with mild or no symptoms due to left-sided valvular heart disease. Emphasis is placed on evidence from high-impact cardiothoracic surgery and cardiology journals. Clinical guidelines and expert reviews are referenced selectively to provide context, but the analysis is grounded primarily in original clinical data. The objective was to delineate how symptom status and patient age should inform operative timing and prosthesis choice. The initial sections examine decision-making principles applicable to both aortic and mitral valve disease. Subsequent portions of the review focus predominantly on surgical aortic valve replacement (SAVR), with particular attention to long-term prosthesis performance in mildly symptomatic populations. Outcomes are stratified by age group (<50, 50–70, and >70 years) and by underlying valve pathology. By synthesizing high-quality recent evidence, this review seeks to inform clinical practice regarding optimal timing of intervention in early-stage valvular disease.

## 6. Pathophysiology of valve stenosis and regurgitation

**Figure 2:**
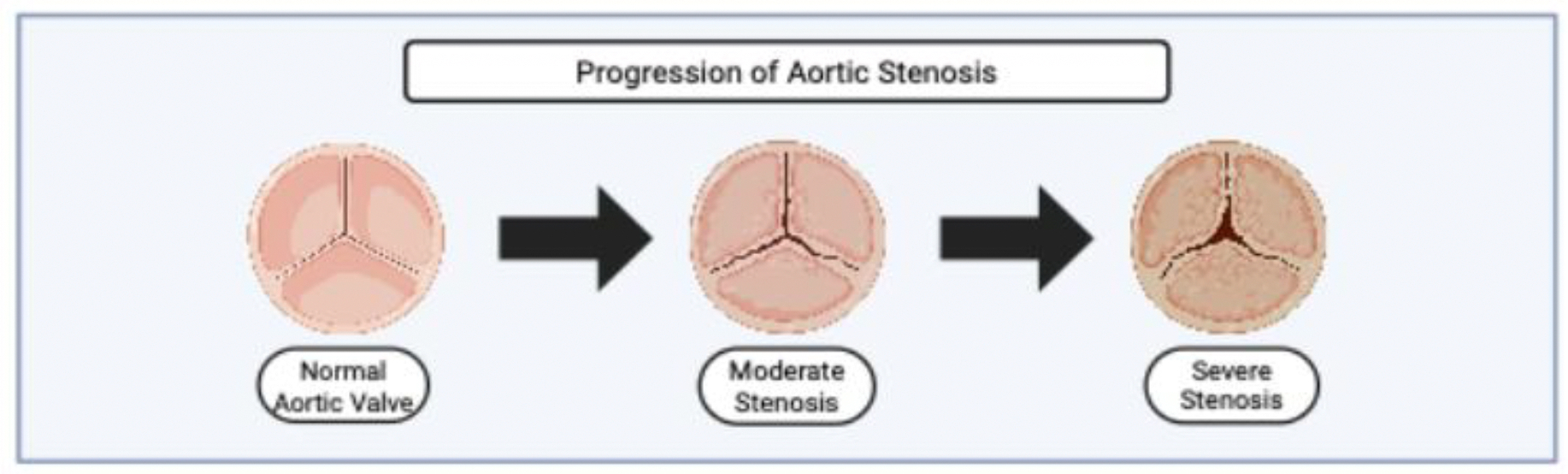
En face superior view of the progression of aortic stenosis, created with BioRender. The illustration shows a normal trileaflet aortic valve (left), followed by progressive cusp thickening and restricted mobility in moderate and severe stenosis. Arrows indicate the continuum of valvular degeneration as leaflet calcification worsens and the orifice narrows.

Valvular heart disease disrupts normal cardiac hemodynamics and imposes pressure or volume overload on affected chambers (Figure 2). In developed countries, aortic stenosis most commonly results from calcific degeneration or bicuspid morphology, leading to progressive leaflet thickening and reduced mobility [6, 7]. This calcific process is driven by endothelial injury, chronic inflammation, and osteogenic transformation of valvular interstitial cells, ultimately producing left ventricular (LV) pressure overload and concentric hypertrophy [14, 15]. In contrast, chronic aortic regurgitation arises from leaflet degeneration or aortic root dilation, resulting in diastolic backflow and progressive LV volume overload (Figure 2). Over time, this leads to eccentric chamber dilation and eventual systolic dysfunction [1,6]. Mitral stenosis (MS), most often rheumatic in origin, causes obstruction of LV inflow, elevating left atrial (LA) pressures and inducing pulmonary venous hypertension and right ventricular strain [6, 7]. Mitral regurgitation, typically secondary to degenerative prolapse or functional remodeling, results in LA and LV volume overload, with atrial dilation and eccentric LV hypertrophy [2, 5, 6]. These hemodynamic changes form the basis of symptom development and long-term morbidity if left untreated [16].

## 7. Functional classification and guidelines for surgical intervention

Symptom severity in valvular heart disease is graded using the New York Heart Association (NYHA) functional classification system, which ranges from Class I, indicating no limitation of physical activity, to Class IV, characterized by symptoms at rest. Class II corresponds to symptoms with ordinary exertion, while Class III reflects significant limitation with minimal activity. Even mild functional impairment, as defined by Class II status, is prognostically relevant in patients with severe valve lesions and is associated with reduced exercise tolerance and increased risk of adverse cardiovascular events [15, 17]. Current management guidelines from the ACC/AHA and ESC recommend surgical intervention for patients with severe symptomatic valvular disease and for select asymptomatic individuals with early evidence of ventricular decompensation or adverse remodeling [6, 7]. Specific procedural thresholds, lesion-specific triggers, and guideline comparisons are discussed in detail in a later section. In all forms of VHD, the optimal timing of intervention is intended to prevent irreversible myocardial remodeling. Once pathological changes such as myocardial fibrosis or impaired contractility have developed, postoperative outcomes decline substantially despite successful valve replacement [6, 7, 10].

## 8. Surgical risk assessment

When surgical intervention is considered, validated risk prediction models play a central role in guiding clinical decision-making. The Society of Thoracic Surgeons Predicted Risk of Mortality (STS-PROM) and the European System for Cardiac Operative Risk Evaluation II (EuroSCORE II) are the most widely used tools, incorporating patient-specific variables such as age, comorbid burden, and procedural characteristics. These models have demonstrated reliable predictive performance. In one study, EuroSCORE II yielded an area under the receiver operating characteristic curve between 0.79 and 0.83 for predicting 30-day and in-hospital mortality in a Taiwanese cohort [18]. Comparative analyses suggest that STS-PROM may outperform EuroSCORE II in calibration and discriminative capacity, particularly among patients undergoing aortic valve replacement [19]. In clinical practice, patients classified as low risk (STS <4% or low EuroSCORE II) are typically suitable surgical candidates. Conversely, high predicted risk (STS >8% or equivalent) often prompts consideration of less invasive alternatives, such as transcatheter aortic valve replacement (TAVR) or transcatheter mitral valve repair (e.g., MitraClip). Beyond algorithmic scores, frailty and other individualized factors must be integrated into decision-making. In patients with prohibitive operative risk, surgery is generally avoided unless emergent intervention is necessary [20, 21].

## 9. Timing of surgical valve replacement in mildly symptomatic patients

### 9.1. Current guidelines and thresholds for intervention

Current valve surgery guidelines emphasize timely intervention to prevent irreversible ventricular remodeling or dysfunction [6, 7]. In aortic stenosis, the presence of symptoms in patients with severe AS is a Class I indication for aortic valve replacement, as is a reduction in left ventricular ejection fraction (LVEF) below 50% in asymptomatic individuals. Patients with severe AS and peak aortic jet velocity ≥5.0 m/s or mean transvalvular gradient ≥50 mmHg are eligible for early AVR under a Class IIa recommendation, as are those who develop symptoms or hypotension during exercise testing. A rapid increase in peak velocity of ≥0.3 m/s per year may support intervention under a Class IIb indication [7]. In aortic regurgitation, surgical intervention is indicated in all patients with severe AR who develop symptoms or in whom LVEF declines into the mid-50% range, corresponding to a Class I recommendation. Asymptomatic patients with progressive left ventricular dilation or an LVEF approaching the lower limits of normal (55–60%) may be considered for earlier intervention. Observational studies suggest that outcomes worsen once LV end-systolic dimension exceeds approximately 45–50 mm, supporting this threshold as a useful marker for surgical timing [1, 6, 7, 22]. For degenerative mitral regurgitation, current guidelines recommend surgical intervention once symptoms appear or LV function declines, corresponding to a Class I indication when the LVEF is between 30–60% or the LV end-systolic dimension exceeds 40 mm [6, 7, 23]. In asymptomatic patients with preserved LV function (LVEF >60%, LVESD <40 mm), early mitral valve repair is supported as a Class IIa recommendation if a durable repair is likely. This approach is backed by observational studies showing improved long-term outcomes with early surgery over watchful waiting in select patients [10]. Once LV dysfunction or chamber enlargement becomes evident, outcomes decline, reinforcing the importance of early referral. In clinical practice, centers with high repair success rates may pursue early intervention in select asymptomatic patients, balancing procedural risk with the opportunity to preserve ventricular performance [6, 7, 11].

## 10. Recent studies and trials on early vs. delayed surgery

### 10.1. Aortic Stenosis (AS) trials and meta-analyses

**Figure 3:**
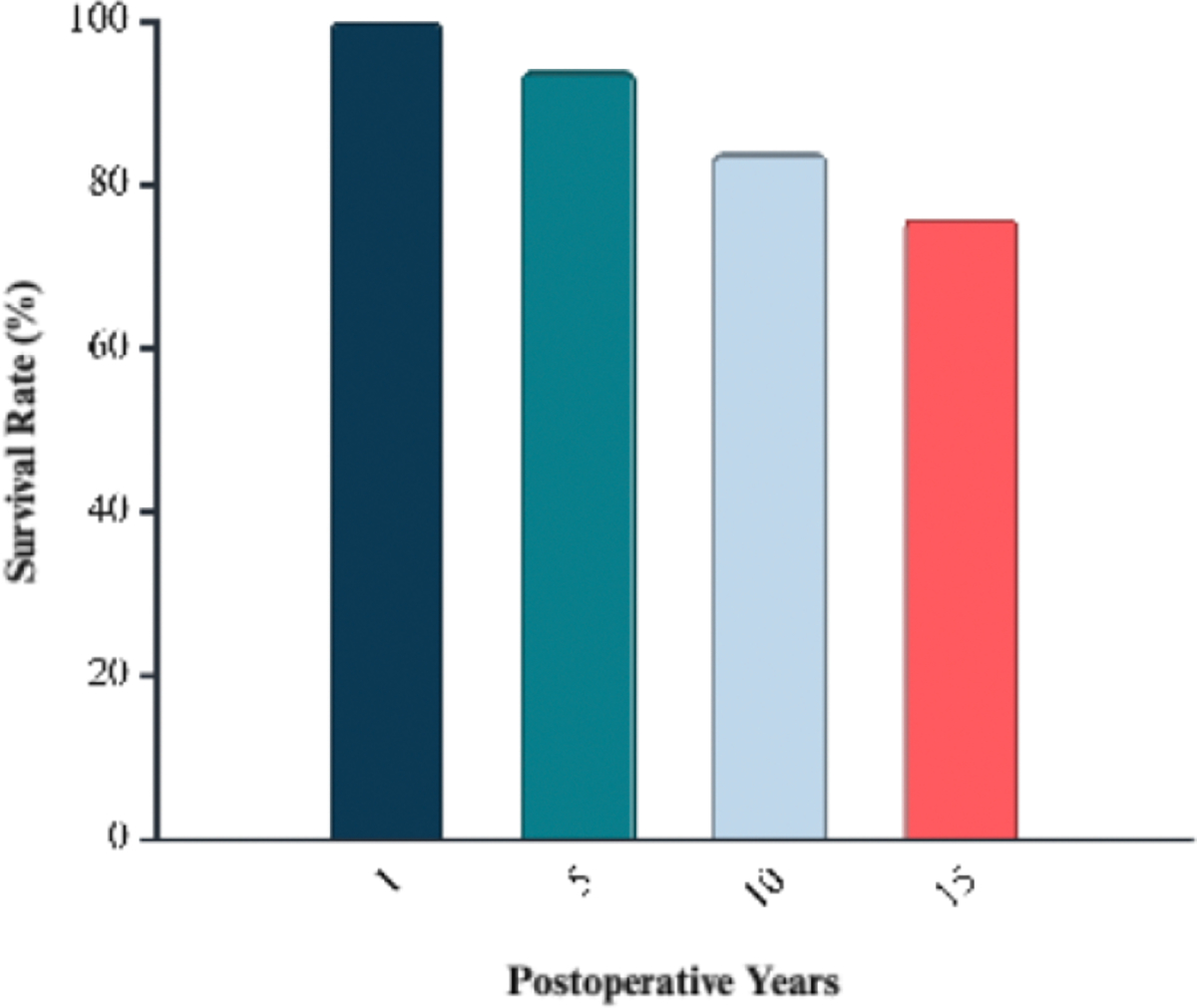
Postoperative survival after surgical aortic valve replacement (SAVR) in asymptomatic patients with severe aortic stenosis. Survival at 1, 5, 10, and 15 years was 100%, 94%, 84%, and 76%, respectively. The Data were reviewed and the % survival rate after SAVR 1-to-15 years postoperatively was calculated and based on the published data by Javadikasgari et al. [25].

Multiple recent trials have evaluated early SAVR compared to conservative management in asymptomatic patients with severe to very severe AS. The landmark RECOVERY trial was the first randomized controlled study to evaluate early SAVR in patients with very severe AS who were strictly asymptomatic, as confirmed by clinical assessment and exercise testing. Patients were randomized to undergo early SAVR or receive conservative care [8]. Early surgery resulted in no operative mortality and significantly fewer primary endpoint events: only 1 of 73 patients in the early-SAVR group met the composite endpoint versus 11 of 72 in the surveillance group (hazard ratio ≈ 0.09) [8]. All-cause mortality was also substantially lower with early surgery (7% vs. 21%; hazard ratio ≈ 0.33) [8]. The AVATAR trial (2022) expanded on these findings in a broader cohort of patients with asymptomatic severe AS. In this multicenter randomized study, early SAVR significantly reduced the composite of all-cause mortality and heart failure hospitalization compared with watchful waiting [9]. Building on both RECOVERY and AVATAR, a 2025 meta-analysis of four randomized controlled trials (n ≈ 1,400) confirmed that early aortic valve intervention, via either SAVR or TAVR, was associated with significantly fewer unplanned cardiovascular or heart failure hospitalizations (hazard ratio ≈ 0.40) and a lower incidence of stroke (hazard ratio ≈ 0.62) over a median follow-up of four years. However, differences in all-cause and cardiovascular mortality did not reach statistical significance [24]. Additional long-term data further support the benefits of early intervention. A 2024 cohort study of asymptomatic patients undergoing SAVR reported postoperative survival rates of 100%, 94%, 84%, and 76% at 1, 5, 10, and 15 years, respectively (Figure 3). These outcomes surpassed those of age- and sex-matched population controls [25]. Notably, patients with moderate or severe preoperative left ventricular hypertrophy experienced significantly worse long-term survival and persistent diastolic dysfunction despite surgery. These results suggest that irreversible myocardial remodeling may begin prior to symptom onset and that postponing intervention could diminish the potential benefit of surgery [25].

### 10.2. Mitral regurgitation (mr) studies

**Figure 4:**
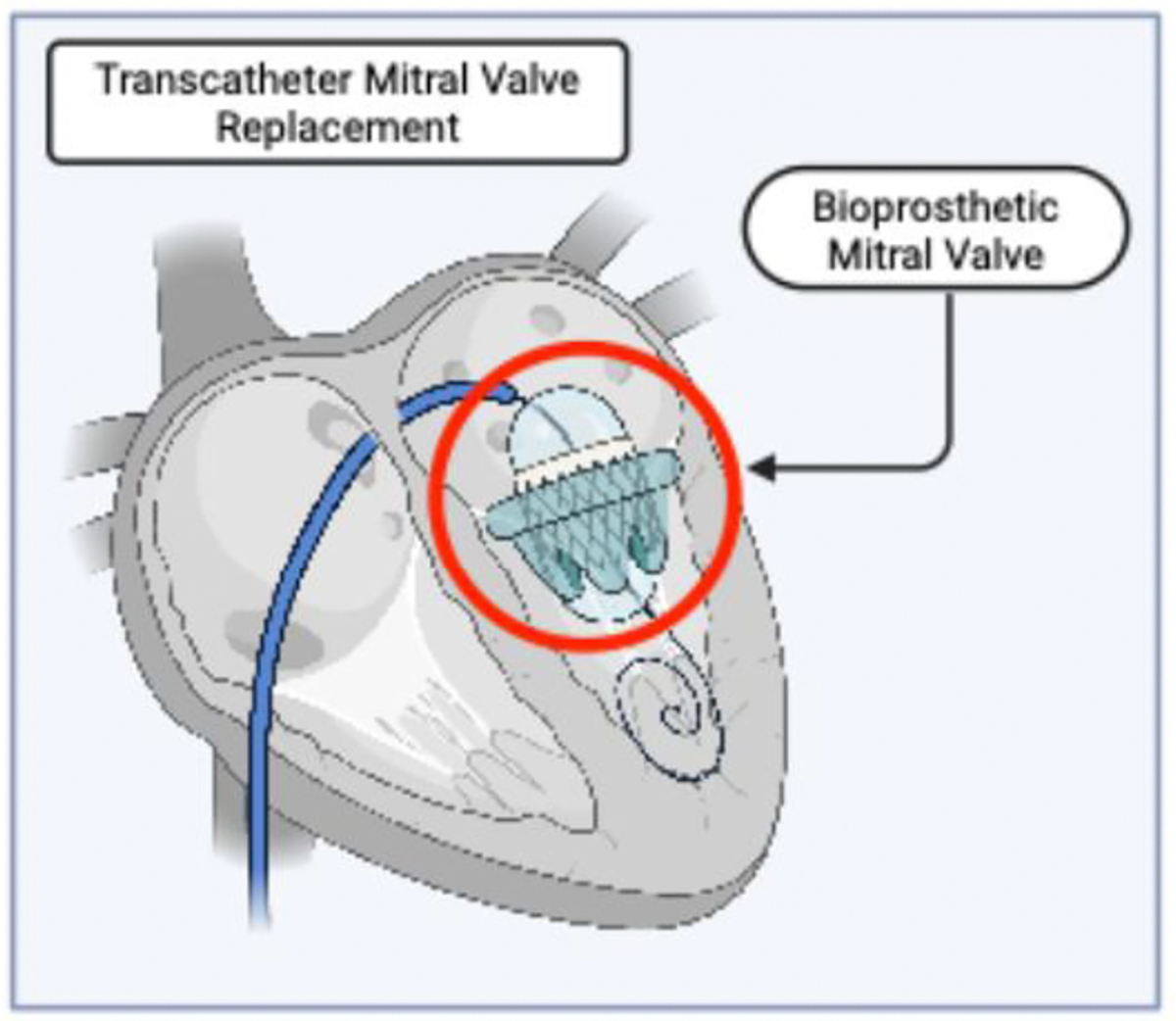
Cross-sectional illustration of transcatheter mitral valve replacement (TMVR), created with BioRender. A balloon-expandable bioprosthetic mitral valve is shown positioned within the native annulus, with the delivery balloon still inflated during the deployment phase of valve implantation.

To date, no randomized controlled trials have directly compared early versus delayed surgery in patients with primary MR. Current evidence is derived primarily from observational cohort studies [11]. These consistently demonstrate that early mitral valve (MV) surgery, typically repair, in asymptomatic patients with severe degenerative MR is associated with excellent clinical outcomes. Operative mortality is low, and long-term survival remains favorable [10]. In one series of 145 asymptomatic individuals (mean age 60) who underwent MV surgery, the 10-year survival rate approached 91%, accompanied by high quality-of-life scores (median Kansas City Cardiomyopathy Questionnaire = 100) and minimal postoperative regret [26]. In contrast, deferral of surgery until symptoms develop or left ventricular dysfunction becomes apparent is associated with an increased risk of adverse outcomes. Clinical guidelines emphasize that delays beyond established thresholds for LV ejection fraction or end-systolic dimension are linked to worse prognosis [6,7]. While a strategy of watchful waiting may be appropriate in select cases with high operative risk or borderline indications, referral for early intervention is generally favored in high-volume centers where durable MV repair can be reliably performed with minimal perioperative risk [10, 11]. Operative strategy also influences long-term outcomes. Bioprosthetic mitral valve replacement is a reasonable alternative in patients who are not suitable candidates for repair and may be performed using a transcatheter approach in select cases (Figure 4). In a 10-year follow-up of a Medicare cohort receiving porcine bioprosthetic valves, structural valve deterioration was infrequent and reintervention rates remained within acceptable limits, supporting the use of bioprostheses in appropriately selected patients [27]. However, mitral valve repair remains the preferred approach given its superior preservation of LV function and lower rates of valve-related morbidity over time.

Importantly, favorable outcomes following MV repair have been reported in both high- and low-volume institutions, with no significant differences in operative mortality or long-term survival based on surgeon experience alone [28]. Additionally, minimally invasive techniques, including robotic-assisted and mini-thoracotomy approaches, have demonstrated equivalent safety and efficacy compared to conventional sternotomy. These techniques also offer benefits such as reduced length of stay and more rapid postoperative recovery [29,30].

### 10.3. Aortic regurgitation (ar) observations

In chronic aortic regurgitation, evidence regarding timing of surgery is primarily derived from observational studies. A 2024 Japanese cohort of asymptomatic patients with severe AR and preserved left ventricular ejection fraction found that a strict strategy of watchful waiting was generally safe. Over a median follow-up of approximately three years, cardiac mortality was low, and overall survival was comparable to that of an age- and sex-matched general population [31]. However, this study identified a preoperative left ventricular end-systolic diameter (LVESD) threshold of approximately 45 mm beyond which postoperative outcomes were significantly worse. An LVESD threshold of 45–50 mm may represent an inflection point beyond which outcomes worsen, supporting its use in surgical decision-making [31]. While randomized controlled trials are lacking, ongoing investigations such as the ELEANOR trial aim to further evaluate the benefits of early surgery in asymptomatic AR [32]. An additional consideration involves the management of mild or moderate aortic stenosis identified incidentally during cardiac surgery for another indication. In a 2024 cohort study of patients undergoing septal myectomy for obstructive hypertrophic cardiomyopathy, those who underwent concurrent aortic valve decalcification or valve replacement had significantly lower rates of subsequent aortic valve reintervention compared to patients whose valves were not treated [33]. Hemodynamic outcomes and long-term survival were similar across all groups, and perioperative morbidity was not significantly increased. Although these results pertain to a specific surgical population, they support the broader principle that addressing borderline or subclinical valve disease at the time of other cardiac procedures may reduce future progression and avoid the need for subsequent interventions [33].

### 10.4. Imaging, stress testing, and biomarkers

Advanced imaging and biomarker assessment are increasingly employed to refine surgical timing in asymptomatic or mildly symptomatic valvular disease. In aortic stenosis, left ventricular global longitudinal strain (GLS) measured by echocardiography has emerged as a sensitive indicator of subclinical dysfunction. A meta-analysis of 1,512 asymptomatic AS patients found that impaired GLS (worse than −15%) was significantly associated with increased mortality and adverse cardiovascular events, independent of left ventricular ejection fraction [34]. Strain imaging may therefore help identify candidates for earlier intervention, even when conventional measures such as LVEF remain within normal limits.

Exercise echocardiography provides additional prognostic insight. A recent prospective study of asymptomatic patients with severe AS demonstrated that serial annual exercise testing safely revealed symptom onset or abnormal blood pressure responses, which prompted timely referral for aortic valve replacement. Notably, no sudden cardiac deaths occurred during testing, underscoring the safety and utility of this approach in surveillance [35].

Biomarkers also play a role in identifying high-risk patients. B-type natriuretic peptide (BNP), a marker of myocardial strain, is predictive of adverse outcomes in severe AS. Guidelines now recommend considering AVR when BNP levels exceed three times the normal range, even in the absence of overt symptoms [36]. Elevated BNP reflects increased wall stress and correlates with disease severity, providing a biochemical signal for impending decompensation [36]. Similar principles apply in mitral valve disease. In asymptomatic severe mitral regurgitation, reductions in GLS and rising natriuretic peptide levels are under investigation as early indicators of LV impairment [10, 11]. Progressive chamber enlargement on serial imaging remains an established trigger for surgery in both MR and AR.

In a retrospective cohort of 673 asymptomatic patients with moderate–severe AR and preserved LVEF, subclinical markers such as GLS worse than −15%, indexed end-systolic volume ≥45 mL/m^2^, or LVEF <60% were each associated with increased all-cause mortality. The presence of all three parameters conferred a more than fivefold increased risk (HR 5.46; P < 0.001) [37]. These results support the integration of quantitative imaging and biomarkers into surveillance algorithms for chronic AR. Collectively, these modalities extend risk stratification beyond symptoms and resting LVEF. In asymptomatic or borderline-symptomatic patients, their incorporation can guide earlier surgical referral before irreversible ventricular remodeling occurs [10].

## 11. Outcomes: mortality, heart failure, and long-term function

Recent trials provide robust longitudinal outcome data. In AS, the RECOVERY trial demonstrated that early SAVR markedly improves survival. At four years, cumulative survival was approximately 96% in the early-surgery group versus 88% in the surveillance group [8]. Meta-analysis likewise indicates that early aortic valve intervention reduces the risk of heart failure hospitalization by nearly half [24]. Long-term outcomes following early mitral valve repair are similarly favorable. One cohort study of asymptomatic patients reported excellent functional outcomes, with sustained freedom from heart failure symptoms and low reoperation rates. Nearly all patients expressed high satisfaction and a willingness to undergo surgery again, underscoring the durable benefit and quality-of-life improvement associated with early mitral intervention [26]. Comparatively limited high-quality data exist on delayed surgery, but available evidence suggests that late referral is associated with higher rates of postoperative left ventricular dysfunction and heart failure [11, 23]. In AS, long-term follow-up data from a single-center cohort reported that survival following early SAVR surpassed that of age- and sex-matched population controls. However, outcomes were significantly worse among patients with preoperative LV hypertrophy or diastolic dysfunction, suggesting that irreversible myocardial remodeling may begin prior to symptom onset [25]. Crucially, the risks of waiting must be weighed against the operative risks of early intervention. Contemporary SAVR is associated with low perioperative mortality, often under 1–2% in elective cases [18,19]. Delaying intervention increases the risk of permanent myocardial damage. In the RECOVERY trial, 14% of patients in the conservative arm experienced sudden cardiac death by eight years, whereas no such events occurred in the early surgery group [8]. Conversely, early surgery in carefully selected asymptomatic patients confers low morbidity and durable valve performance, particularly in experienced centers [9, 24]. Collectively, these findings support timely surgical intervention in patients with severe valvular disease, even in the absence of overt symptoms. Early surgery is associated with improved survival, fewer heart failure events, and better long-term function, while delays risk structural deterioration and diminished postoperative benefit [8,11,17,24–26].

## 12. Valve selection: mechanical vs. bioprosthetic valves

### 12.1. Factors affecting valve choice age, life expectancy, and lifestyle impact

Valve selection in surgical aortic valve replacement is primarily influenced by patient age, life expectancy, and anticipated durability requirements. In individuals under 65 years of age with projected survival exceeding 10 to 15 years, mechanical prostheses are generally preferred due to their superior structural longevity and lower incidence of reoperation [38–41]. In contrast, bioprosthetic valves are more frequently selected in older patients or those with contraindications to anticoagulation, offering the benefit of reduced thromboembolic risk and obviating the need for lifelong warfarin therapy [12, 39, 42]. A large Society of Thoracic Surgeons (STS) registry analysis of patients aged 40 to 75 demonstrated a significant long-term survival advantage with mechanical valves, particularly in the younger subset [38]. Similarly, a retrospective cohort study of patients aged 50 to 70 reported higher all-cause mortality with bioprostheses (HR 1.39), suggesting a clinically relevant impact of structural valve degeneration in this age group [39]. However, the survival benefit of mechanical valves diminishes in older patients, where competing non-cardiac mortality limits the relevance of late valve failure [12, 13, 43]. Prosthesis durability remains a critical determinant. Mechanical valves reliably function for decades but necessitate continuous anticoagulation, increasing the risk of major bleeding. Bioprostheses, while increasingly robust, remain susceptible to structural deterioration, especially in younger patients [39, 42, 44, 45]. Recent data indicates that up to one-third of bioprosthetic valves may fail within 10 to 15 years in patients under 60, with earlier onset of structural valve degeneration directly correlated with younger age at implantation [42, 45]. In a cohort of patients aged ≤65 undergoing SAVR, freedom from structural valve deterioration was lower among bioprosthesis recipients, despite acceptable overall survival, underscoring the durability limitations of tissue valves in younger adults [41]. Contemporary practice increasingly incorporates patient preferences into prosthesis selection. Registry data and patient surveys indicate that many individuals prioritize freedom from anticoagulation, even when informed of the elevated risk of future reintervention [6,46]. This has reinforced the role of shared decision-making in valve strategy, particularly when guideline-recommended options differ minimally in survival outcomes. Anticipated adherence to anticoagulation, reproductive planning, occupational considerations, and tolerance for potential reoperation are among the key factors that must be explicitly discussed [6, 44, 47].

### 12.2. Technical Considerations and Risk Profiling

Anatomical and technical considerations also modulate prosthesis choice. In patients with small aortic annuli, aortic root enlargement procedures such as the Nicks or Manouguian technique permit implantation of larger valves, minimizing the risk of prosthesis–patient mismatch. Studies have demonstrated that these approaches achieve favorable hemodynamic profiles without increased perioperative risk compared to full root replacement[48, 49]. In contrast, uncorrected mismatch has been associated with impaired left ventricular remodeling and reduced survival, as shown in pooled analyses from the PARTNER trials [50].

Early data from a five-year follow-up of bicuspid aortic valve replacement using RESILIA tissue suggests stable hemodynamic function and low rates of structural valve degeneration in this population, though extended durability remains under investigation [51]. Moreover, valve choice must account for the likelihood of reintervention over a patient’s lifetime, as even modern surgical bioprostheses typically exhibit durability limits of 12 to 15 years [45].

Procedural risk profiles must also inform valve strategy. For instance, analysis of a trans-Pacific registry revealed comparable one-year mortality between men and women after TAVR, but significantly higher stroke rates among female patients, highlighting the importance of incorporating sex-based risk stratification into valve selection discussions[47]. In scenarios where clinical outcomes are projected to be similar across prosthesis types, patient-defined priorities often determine the final treatment plan. Aligning prosthesis choice with individualized goals enhances patient satisfaction and ensures that therapeutic decisions reflect both clinical evidence and long-term quality-of-life considerations.

### 12.3. Bleeding and Thromboembolism Risk

Mechanical prostheses necessitate indefinite anticoagulation with warfarin, introducing a sustained risk of major bleeding and imposing significant limitations on daily life. In a cohort of middle-aged patients undergoing AVR, mechanical valves were associated with more than double the incidence of hemorrhagic and thromboembolic events compared to bioprosthetic valves [39]. Maintenance of therapeutic INR requires regular monitoring and is susceptible to fluctuations from dietary changes, concurrent medications, and procedural interruptions. Warfarin’s teratogenicity also renders it contraindicated during pregnancy, posing a particular challenge for reproductive-aged patients considering mechanical valve implantation [44]. Insufficient anticoagulation markedly increases the risk of valve thrombosis and systemic embolism in mechanical valve recipients. Although warfarin mitigates this risk effectively, the requirement for lifelong anticoagulation remains a major limitation of mechanical prostheses. In contrast, patients receiving bioprosthetic valves typically require only a short postoperative anticoagulation period, commonly 3 to 6 months, after which most can be transitioned to antiplatelet monotherapy with aspirin [42]. Despite therapeutic anticoagulation, mechanical valves carry a low but ongoing risk of thromboembolic stroke. Bioprosthetic valves, while less durable, have been associated with lower long-term stroke incidence. Procedural risk may also vary by sex. A trans-Pacific registry study demonstrated comparable 1-year mortality between men and women following TAVR, but stroke occurred more frequently in women, underscoring the importance of incorporating sex-specific outcome data into procedural planning [47]. Efforts to reduce bleeding risk through modified anticoagulation targets have yielded encouraging results in select populations. The PROACT Aortic trial investigated lower-intensity anticoagulation (INR 1.5–2.0) in patients with On-X mechanical valves and found a significant reduction in major bleeding without an increase in thromboembolic events [52]. The 2023 PROACT Mitral trial evaluated low-dose versus standard-dose warfarin in mechanical mitral valve recipients. While the trial did not demonstrate formal noninferiority, stroke and bleeding rates were similar between groups, suggesting potential applicability in specific clinical settings [53]. These trials indicate that lower-intensity warfarin regimens may reduce bleeding risk but do not eliminate the need for lifelong anticoagulation in patients with mechanical valves. The balance between minimizing thromboembolic events and avoiding bleeding complications remains central to prosthesis selection. For patients at elevated bleeding risk with limited access to anticoagulation monitoring, or with future pregnancy plans, bioprosthetic valves may be more appropriate. Conversely, in younger patients who can maintain stable INR and are willing to accept the lifestyle adjustments required, mechanical valves offer unmatched durability.

## 13. Evidence comparing valve types in various populations

### 13.1. Outcomes by age group (<50, 50–70, >70 years)

Age remains one of the most influential factors in prosthetic valve selection, with long-term outcomes varying significantly across different decades of life. In younger patients under 50, mechanical prostheses continue to demonstrate superior durability and survival. A multicenter analysis of AVR recipients under 60 found that mechanical valves were associated with improved 15-year survival compared to bioprostheses, particularly in the 50–60 age range [38, 54]. Similarly, a large multicenter analysis reported that among patients aged 40 to 60 years, mechanical valve recipients had higher survival rates and markedly fewer valve-related complications [38]. Additional studies suggest mechanical valves may reduce the risk of reoperation and thromboembolic events in this population [44].

In patients aged 50 to 70 years, survival differences between valve types appear less pronounced. A 2022 meta-analysis found that bioprosthetic AVR was associated with higher all-cause mortality in patients under 70 (pooled HR 1.22), though bleeding risk was higher with mechanical prostheses [42]. A large national cohort study similarly observed no statistically significant difference in adjusted long-term survival between mechanical and bioprosthetic AVR in this age group, although mechanical valves remained associated with a lower incidence of reoperation [12, 41]. A separate multicenter analysis reported that patients over 50 receiving bioprosthetic valves experienced higher reintervention rates despite comparable survival [13]. In a retrospective cohort of patients aged 50 to 70 years, bioprosthetic valves were associated with increased all-cause mortality and more frequent structural valve degeneration [39] Data from the Avalus bioprosthesis trial further support this, showing favorable five-year outcomes in both ≤65 and >65 subgroups, with low structural valve degeneration and excellent hemodynamic performance [41]. Among patients over 70, the survival advantage of mechanical prostheses diminishes further, as competing non-cardiac mortality becomes more prominent. In a propensity-weighted analysis of patients ≥70 years, long-term survival was similar between valve types, but mechanical valves carried a higher incidence of major bleeding and thromboembolic complication. This aligns with broader registry trends favoring bioprosthetic valves in older adults with shorter life expectancy or increased bleeding risk [43, 54]. Together, these data reinforce an age-stratified approach to prosthesis selection: mechanical valves are generally preferred in patients under 60 who can tolerate anticoagulation, while bioprostheses are favored in older adults where reintervention is less likely to occur within the valve’s lifespan. In the intermediate 50–70 age group, individual preferences and comorbidities often determine the optimal valve strategy.

### 13.2. Reoperation rates

Structural valve degeneration remains the predominant cause of reintervention following surgical bioprosthetic valve replacement, particularly in patients with longer projected survival. The risk of reoperation is inversely related to age at implantation, with younger individuals experiencing more rapid degeneration. As previously noted, in patients under 70 years, bioprosthetic valves are associated with a markedly higher likelihood of reintervention, with pooled hazard ratios exceeding 3.0 in meta-analysis [42], and a multicenter cohort study reporting more than a sixfold increase in reoperation risk compared to mechanical valves [39].

Degenerative failure is not limited to older-generation prostheses. Subclinical structural valve deterioration has been identified in recipients under 60, often progressing to hemodynamically significant dysfunction within a decade [55]. Comparative analyses of specific bioprosthetic valve types (e.g., Trifecta, Mitroflow) report significantly lower freedom from reoperation than with platforms such as the CE-Perimount [56].

Sex-based differences in durability have also been observed. Among younger women undergoing AVR, the reintervention rate was 8.8% for bioprostheses versus 1.8% for mechanical valves, despite similar overall survival [44]. Furthermore, even with newer-generation devices, patients below 65 years of age often require reintervention within 10 to 15 years [41, 45]. In contrast, mechanical prostheses are rarely subject to structural failure, although the need for lifelong anticoagulation presents its own risks and lifestyle considerations. While valve-in-valve transcatheter procedures offer a less invasive reintervention strategy, procedural feasibility may be limited by annular anatomy, and the long-term durability of sequential implants remains uncertain, particularly in recipients with small annuli or narrow primary prostheses [57]. Structural deterioration is also more frequently observed when bioprosthetic valves are implanted in the mitral position, further compounding reintervention risk among patients with longer life expectancy. In preoperative planning, integrating patient age and valve position remains essential for optimizing valve durability and minimizing the need for future reintervention [58].

## 14. Innovations in valve technology and their impact

### 14.1. Sutureless and rapid-deployment valves

Newer surgical bioprostheses, such as the Perceval and Intuity valves, utilize sutureless or rapid-deployment technology to facilitate implantation and reduce operative time. Registry analyses indicate comparable 30-day mortality to conventional stented valves, while significantly shortening cardiopulmonary bypass and aortic cross-clamp durations [59]. In a large Society of Thoracic Surgeons (STS) database study of approximately 17,700 patients, sutureless valves were associated with an 18-minute reduction in cross-clamp time and were more frequently used in minimally invasive procedures, with no difference in in-hospital mortality (3.1% vs 3.1%) compared to conventional valves [59]. However, sutureless valves carried higher pacemaker implantation rates (≈11% vs 5%)[59].

Mid-term outcomes across multiple centers have shown satisfactory results, with reported survival approaching 84% at five years and stable hemodynamic profiles [60]. Additional analyses have demonstrated low stroke and mortality rates at midterm follow-up [61]. These valves may be particularly beneficial in high-risk patients or those undergoing minimally invasive surgery, offering improved intraoperative efficiency without compromising short-term safety. Nevertheless, they do not address the intrinsic limitations of bioprosthetic valves, particularly the risks of structural valve degeneration and limited long-term durability.

Expanding on these innovations, totally endoscopic and right anterior thoracotomy-based surgical aortic valve replacement techniques have become feasible in carefully selected patients. These minimally invasive approaches offer a reduced surgical footprint and have demonstrated favorable early outcomes in institutional series [62, 63]. Collectively, these advances in surgical technology have broadened the applicability of AVR and may support earlier intervention in patients previously considered borderline surgical candidates.

### 14.2. TAVR vs. SAVR in low-risk patients

The emergence of transcatheter aortic valve replacement has transformed the landscape of aortic valve therapy and now plays a central role in prosthesis selection. In patients deemed low surgical risk, randomized trials have demonstrated that TAVR achieves comparable outcomes to SAVR at midterm follow-up. The PARTNER 3 trial, which enrolled low-risk patients with severe aortic stenosis, reported no significant differences in the composite endpoint of death, stroke, or rehospitalization at 5 years between TAVR (22.8%) and SAVR (27.2%; p=0.07) [64]. Rates of stroke, all-cause mortality, and bioprosthetic valve failure (~3–4%) were similar between the two groups, supporting the use of TAVR as a viable alternative to SAVR in appropriately selected elderly low-risk patients [64]. Despite these promising results, concerns persist regarding TAVR durability in younger patients and the implications for long-term valve performance. A 2023 meta-analysis of low-risk populations found no significant differences in reintervention or all-cause hospital readmission between TAVR and SAVR. However, TAVR was associated with a significantly higher incidence of permanent pacemaker implantation and elevated midterm all-cause mortality [65]. A 2025 prospective cohort study further reinforced these concerns, demonstrating that although 5-year mortality and stroke rates remained comparable, TAVR continued to carry a greater risk of pacemaker implantation, even among well-selected low-risk patients [66]. Furthermore, when surgical intervention is required after a prior TAVR, the associated perioperative risks are markedly increased compared to primary SAVR, highlighting the importance of initial treatment strategy in younger or lower-risk patients with longer anticipated survival [67]. In clinical practice, these risks have contributed to a pattern in which TAVR is favored in older patients with shorter life expectancy, while SAVR remains the preferred approach in younger individuals where long-term durability and surgical reintervention profiles are more favorable [67].

### 14.3. Valve-in-valve (ViV) considerations for future reinterventions

The feasibility of transcatheter valve-in-valve (ViV) replacement for failed surgical bioprostheses is an important consideration influencing prosthesis selection. Patients undergoing bioprosthetic surgical aortic valve replacement may later be eligible for ViV transcatheter aortic valve replacement in the event of structural valve degeneration, thereby potentially avoiding reoperation. Outcomes associated with ViV TAVR have been clinically favorable. In a large national registry analysis, ViV TAVR was associated with significantly lower in-hospital mortality compared to redo surgical AVR (odds ratio, 0.42), with comparable rates of stroke, pacemaker implantation, and other short-term complications [68]. These results demonstrate the viability of ViV as a lower-risk, catheter-based alternative to repeat sternotomy in appropriately selected patients. The availability of ViV techniques has shifted decision-making in favor of bioprosthetic valves for certain patients, particularly when long-term management strategies are considered. However, ViV procedures may be limited by anatomical constraints, such as small annular size or suboptimal initial valve positioning, which can complicate future device deployment and reduce effective orifice area and hemodynamic performance [68]. These factors underscore the importance of careful valve sizing and surgical planning during the index operation. By contrast, surgical reintervention following failed TAVR remains technically challenging and is associated with elevated perioperative risk, prolonged cardiopulmonary bypass times, and difficulties related to stent frame removal or interference [67]. These limitations reinforce the need for thoughtful prosthesis selection at the initial procedure, particularly in younger or lower-risk patients who are more likely to require future intervention.

## 15. Clinical decision-making and future directions

### 15.1. Balancing surgical risk and disease burden

The decision to proceed with surgery in patients exhibiting mild symptoms requires careful assessment of operative risk in relation to the severity of valvular disease and its subclinical myocardial effects. Mild aortic stenosis, while often considered less urgent, is associated with adverse outcomes. A longitudinal study of over 700 patients with mild to moderate AS demonstrated a 17-fold increase in the risk of cardiac mortality compared to the general population [69]. Contemporary data indicate that operative mortality for isolated SAVR is frequently below 2%. High-volume centers often achieve results that surpass those predicted by traditional risk models [70]. For instance, in a cohort managed by a multidisciplinary heart team, where the mean EuroSCORE II was 9.4%, the observed 30-day mortality was only 1.7%, markedly lower than the expected range of 5 to 10% [70]. This discrepancy highlights the limitations of established surgical risk calculators such as STS-PROM, which may overestimate perioperative risk in modern practice. Isolated SAVR continues to offer favorable long-term survival, particularly in patients classified as low risk [71]. There is increasing recognition that delaying intervention until severe symptom onset may permit irreversible left ventricular remodeling. Early surgery in clinically stable patients has been associated with improved outcomes [71, 72]. A meta-analysis comparing early SAVR to conservative management in asymptomatic severe AS reported a 50% reduction in all-cause mortality with early intervention [72]. In addition to symptomatic status, objective indicators of disease burden are increasingly used to guide surgical timing. These include elevated biomarkers such as B-type natriuretic peptide and cardiac troponin, impaired global longitudinal strain on echocardiography, progressive valve calcification, and increasing transvalvular gradients [34, 36]. In this context, surgical decision-making incorporates both objective markers of disease severity and individual patient characteristics. For example, a physiologically robust patient with mild dyspnea but early signs of ventricular dysfunction may benefit from earlier intervention, whereas an older patient with multiple comorbidities and minimal symptoms may be more appropriately managed with continued observation [8, 24]. Taken together, these factors highlight the need to weigh procedural risk against the potential for progressive myocardial injury when determining the optimal timing of surgical intervention.

In anatomically suitable patients, aortic valve repair may represent a viable alternative to replacement. A recent multicenter registry study reported superior one-year survival following repair compared to replacement, supporting increased consideration of this strategy in appropriately selected cases [73].

### 15.2. Multidisciplinary heart team and prosthesis selection

Multidisciplinary heart teams composed of cardiologists, cardiac surgeons, imaging specialists, and anesthesiologists are now recommended for nearly all patients with valvular heart disease. These teams facilitate comprehensive assessment of surgical timing and prosthesis selection by integrating clinical risk, anatomical considerations, and patient preferences. Published data indicate that dedicated heart team evaluation improves referral patterns and outcomes. In an institutional registry of 1,004 patients with complex valve pathology, the heart team recommended intervention (surgical, transcatheter, or hybrid) for 80% of cases and conservative management for 20% [70]. Notably, the observed 30-day mortality rate was 1.7%, markedly lower than the 5 to 9% predicted by EuroSCORE II and STS-PROM models. This outcome supports the premise that collaborative decision-making enables safe expansion of surgical or transcatheter therapy to patients who might not otherwise be selected based on risk scores alone [70]. Heart teams also guide prosthesis choice. Although age remains a key determinant, with mechanical valves typically preferred in younger patients and bioprostheses in older patients, individual risk profiles, lifestyle considerations, and long-term goals are increasingly emphasized. Recent evidence supports the use of mechanical valves in select middle-aged patients. A 2023 meta-analysis including over 32,000 AVR patients aged 50 to 70 demonstrated that mechanical valves were associated with superior 10-year survival and fewer valve-related complications compared to bioprosthetic valves, despite a higher incidence of anticoagulation-related events [54]. Conversely, newer bioprostheses may offer improved performance in younger patients. In a prospective registry of 421 patients with a mean age of 53, no cases of stage 3 structural valve deterioration were observed at one year using glutaraldehyde-free prostheses [74]. Similarly, the Resilia bovine pericardial valve demonstrated >98% freedom from severe structural degeneration at five years in younger populations [75]. Although mechanical valves carry an ongoing annual risk of stroke and bleeding of approximately 1%, they offer long-term durability that may be preferred by younger patients who are willing and able to maintain lifelong anticoagulation. For older individuals or those with contraindications to anticoagulation, bioprosthetic valves remain a reasonable alternative. As noted in recent reviews, tailoring prosthesis selection to individual patient characteristics represents a paradigm for advancing patient-centered care and future research [54, 71]. In patients with small aortic annuli, root enlargement procedures such as the Nicks or Manouguian techniques allow for implantation of larger prostheses, thereby reducing the risk of prosthesis-patient mismatch [48, 49]. A Society of Thoracic Surgeons database analysis of more than 5,000 patients confirmed that annular enlargement was not associated with increased perioperative mortality, stroke, or pacemaker implantation, supporting its safety in current surgical practice [48]. In a separate institutional analysis, annular enlargement yielded comparable outcomes to full root replacement and favorable hemodynamics, reinforcing its role in managing size-constrained anatomy, particularly in younger patients with longer anticipated survival [49].

### 15.3. Advanced imaging and biomarkers in timing decisions

Emerging diagnostic tools are reshaping how clinicians time surgical intervention in valvular heart disease by revealing subclinical myocardial damage. Among these, strain imaging has gained prominence. Global longitudinal strain, often impaired prior to a decline in ejection fraction (EF), has shown strong prognostic value. A 2022 meta-analysis confirmed that reduced GLS in asymptomatic severe aortic stenosis predicted increased cardiovascular events and mortality, reinforcing its role in identifying early ventricular dysfunction [34]. In practice, a GLS worse than −18% may prompt consideration of earlier AVR even when EF remains within the normal range. Exercise echocardiography remains a practical and safe method for unmasking exertional symptoms or hemodynamic compromise in asymptomatic patients with aortic stenosis. A prospective study of 196 such patients demonstrated that serial exercise stress echocardiography (performed annually) identified significant pathologic findings, such as symptom emergence or abnormal blood pressure response, in approximately 30–50% of cases, prompting elective AVR [35]. No cardiac deaths occurred during testing, underscoring the safety of exercise-based surveillance. These data suggest that stress imaging can guide surgical timing by detecting early physiological deterioration, including excessive gradients or rising pulmonary pressures [35]. Serologic biomarkers further refine risk stratification. B-type natriuretic peptide and N-terminal pro-BNP rise with increasing wall stress and correlate with valvular disease severity. Elevated BNP is now a Class IIa indication for AVR in asymptomatic AS, particularly when levels exceed threefold the upper limit of normal [36]. A comprehensive review has shown that natriuretic peptides consistently predict adverse outcomes and track with disease progression across valve pathologies [36]. High-sensitivity troponin (hs-TnT) is also being investigated as a marker of myocardial injury in AS. A recent study reported that elevated hs-TnT independently predicted major adverse cardiovascular events following AVR, even in patients with preserved EF. When combined with GLS, hs-TnT improved early risk identification, helping to flag patients likely to experience decompensation within six months of surgery [76]. Cardiac magnetic resonance (CMR) imaging offers additional insight into myocardial integrity. Quantitative measures such as T1 mapping and extracellular volume (ECV) fraction allow detection of diffuse interstitial fibrosis, while late gadolinium enhancement (LGE) reveals focal scar. Both markers predict adverse postoperative outcomes, and their presence may justify earlier intervention. In a recent 2025 study, serum transforming growth factor-beta 1 (TGF-β1) levels correlated with CMR-detected myocardial fibrosis in patients with AS, suggesting potential for integrating blood biomarkers with advanced imaging to quantify ventricular remodeling [77]. Computed tomography (CT) is also expanding in use. Aortic valve calcium scoring (CT-AVC) provides objective quantification of stenosis severity, especially in low-flow, low-gradient AS where traditional Doppler criteria may be ambiguous. Extremely high calcium scores can indicate rapid disease progression and guide surgical timing in borderline cases [78, 79]. Taken together, these modalities enable a more individualized approach to surgical timing. Multimodal integration, which includes strain imaging, stress echocardiography, serologic biomarkers, CMR, and CT, offers a comprehensive assessment of disease burden that surpasses symptom status or EF alone. As evidence accrues, future guidelines may more formally incorporate these tools into routine clinical algorithms to optimize surgical outcomes in patients with asymptomatic or mildly symptomatic valvular heart disease [45].

## 16. Gaps in durability data and future directions

Accurately predicting prosthetic valve durability remains a major challenge, particularly in younger patients. Contemporary trials and registry studies demonstrate encouraging short- and mid-term outcomes, but long-term follow-up beyond 10 to 15 years is limited. For example, in patients under 60 years of age, the RESILIA registry reported excellent hemodynamic performance with no structural valve deterioration at 1 year [74], and newer bioprostheses have shown >99% freedom from severe structural valve degeneration (SVD) at 5 years [75]. However, observational data suggest that bioprosthetic failure often accelerates beyond the 10- to 15-year mark. In a population-based cohort of patients under 65 years undergoing AVR between 2003 and 2018, bioprosthetic valves were associated with significantly higher late reoperation rates than mechanical valves, with hazard ratios ranging from approximately 2.5 to 4.5 over long-term follow-up [80]. Moreover, among patients aged 55 to 64 years, late mortality was significantly higher in the bioprosthetic group compared to mechanical valve recipients (HR 1.56) [80]. Bioprosthetic valve recipients under 65 are likely to outlive their initial implant and require reintervention, as indicated by long-term observational data. In contrast, mechanical valves eliminate the risk of SVD but require lifelong anticoagulation and are associated with an ongoing risk of bleeding and thromboembolism [80]. This uncertainty surrounding lifetime valve management underscores the need for extended follow-up of novel prostheses. Longitudinal data beyond 10 years are urgently needed, particularly for recently introduced bioprosthetic platforms. Additionally, clinical trials evaluating strategies such as planned valve-in-valve (ViV) TAVR following initial surgical bioprosthesis are warranted. While ViV procedures have become increasingly feasible, the long-term durability of sequential interventions remains unknown.

In younger patients, where life expectancy often exceeds the expected lifespan of a bioprosthetic valve, future studies should inform recommendations regarding initial prosthesis choice, such as whether to pursue mechanical valve implantation or preserve the option for staged transcatheter reintervention. The role of the multidisciplinary heart team remains central as emerging evidence from ongoing trials, including those evaluating earlier intervention in moderate AS or asymptomatic severe AS, continues to evolve. Guideline development and shared decision-making will increasingly rely on advanced diagnostics and personalized modeling [70, 72]. Ultimately, research efforts should prioritize the development of individualized risk prediction tools, potentially incorporating machine learning and multimodal clinical data, as well as long-term outcome studies of valve performance. These advances will help refine the balance between premature intervention and missed opportunities to prevent irreversible myocardial injury.

## Conclusions

Growing evidence supports timely surgical intervention in patients with severe valvular heart disease who present with mild symptoms. In aortic regurgitation, contemporary data demonstrate that even minor symptoms in older patients are associated with improved outcomes when surgery is pursued. In a cohort of older adults (mean age approximately 75), those undergoing aortic valve replacement had significantly lower all-cause and cardiac mortality than those managed conservatively [1]. Similarly, early surgical repair in young adults with significant AR has been associated with favorable reverse remodeling, with normalization of left ventricular size and function achieved in approximately 65% of patients [81]. For asymptomatic patients with severe mitral regurgitation, elective mitral surgery has produced excellent long-term outcomes, including 91% ten-year survival and preserved quality of life [26]. These findings indicate that delaying surgery until the onset of advanced symptoms may forfeit the optimal therapeutic window for preserving LV function and improving survival.

While the timing of surgery is increasingly guided by subtle symptoms and subclinical LV dysfunction, prosthesis selection continues to depend primarily on patient age, comorbidities, and preferences. Mechanical prostheses are traditionally favored in younger individuals due to their superior durability. A meta-analysis of patients aged 50 to 70 undergoing AVR confirmed that mechanical valves were associated with improved long-term survival and fewer reoperations compared to bioprostheses, despite an increased risk of bleeding events [54]. Longitudinal registry data further support this trend. In one large single-center series, 20-year reintervention rates following mitral valve replacement were substantially lower for mechanical versus bioprosthetic valves (15% vs 59%), with comparable overall survival [82]. Another registry of patients aged 50 to 70 found no overall survival difference by valve type, but landmark analysis showed a late (beyond 12.5 years) survival benefit in favor of mechanical mitral prostheses [46]. These findings align with current guideline recommendations. For patients with anticipated survival exceeding 10 years and no contraindication to anticoagulation, mechanical valves offer enhanced durability and reduced risk of reintervention [54,82]. Nonetheless, modern bioprostheses continue to improve. A glutaraldehyde-free bioprosthetic valve demonstrated >95% freedom from structural valve degeneration at five years [75]. Therefore, tissue valves remain appropriate in older individuals or those seeking to avoid long-term anticoagulation, particularly in the context of expanding transcatheter valve-in-valve capabilities [46,82].

Taken together, the available evidence supports a more proactive surgical approach in mildly symptomatic patients with severe valvular disease. Even minor symptoms or early indicators of ventricular strain, such as borderline LV dilation or impaired longitudinal strain, should prompt timely surgical evaluation, particularly in light of the demonstrated mortality benefit in these populations [1,26]. Best practices include comprehensive preoperative assessment, incorporation of advanced imaging modalities when needed, and multidisciplinary heart team input to guide both timing and prosthesis selection. Mechanical valves remain a durable option for appropriately selected younger patients, while bioprostheses are reasonable in older or anticoagulation-averse individuals, with the understanding that future reintervention may be necessary [54,82]. Shared decision-making remains central to this process and must integrate clinical evidence with patient values and life circumstances.

Despite these advances, several critical gaps persist. No randomized trials to date have directly compared early surgical intervention versus clinical surveillance in mildly symptomatic patients with AR or MR, and current recommendations rely heavily on observational data. Long-term durability of modern bioprosthetic valves beyond 10 to 15 years remains poorly defined [75]. Additionally, early evidence of structural degeneration in transcatheter valves, reported in up to 12% of patients by five years, raises concerns about their suitability in younger populations [83]. Future research should prioritize long-term follow-up of contemporary valve platforms, randomized evaluation of early mitral intervention in moderate or asymptomatic disease, and outcome studies in younger cohorts. In parallel, the development of individualized risk prediction tools, potentially incorporating multimodal imaging and machine learning, may further refine surgical decision-making. Until definitive long-term data are available, clinicians must carefully weigh the risks of delaying surgery against the benefits of early intervention to prevent irreversible myocardial remodeling and optimize long-term outcomes.

## Key Points

- Early surgical intervention in select patients with severe aortic or mitral valve disease and mild symptoms is associated with improved long-term survival and reduced heart failure events, particularly in aortic stenosis.
- Observational evidence supports early surgery in aortic and mitral regurgitation when early ventricular dilation or functional impairment is present, even in the absence of overt symptoms.
- Advanced imaging (e.g., global longitudinal strain) and biomarkers (e.g., BNP, troponin) enhance risk stratification and may help identify patients who benefit from earlier referral.
- Mechanical valves offer superior durability and lower reoperation rates, making them preferable in younger patients without contraindications to anticoagulation.
- Bioprosthetic valves are increasingly used in older patients or those desiring to avoid long-term anticoagulation, though structural degeneration remains a concern in younger recipients.
- Transcatheter valve-in-valve (ViV) procedures expand future options for patients receiving bioprostheses, but anatomical constraints and long-term durability must be considered.
- Multidisciplinary heart team evaluation is essential in determining optimal timing and prosthesis type, particularly for patients with mild or ambiguous symptoms.
- No randomized trials currently exist for early surgery in mildly symptomatic aortic or mitral regurgitation, highlighting the need for further investigation.
